# Epidemiology of Parvovirus B19 Infection In an Italian Metropolitan Area, 2012–2024: COVID‐19 Pre‐Pandemic, Pandemic and Post‐Pandemic Trends

**DOI:** 10.1002/jmv.70296

**Published:** 2025-03-17

**Authors:** Simona Venturoli, Alessia Bertoldi, Elisabetta Manaresi, Tiziana Lazzarotto, Giorgio Gallinella

**Affiliations:** ^1^ Microbiology Unit IRCCS Azienda Ospedaliero‐Universitaria di Bologna Bologna Italy; ^2^ Department of Pharmacy and Biotechnology University of Bologna Bologna Italy; ^3^ Department of Medical and Surgical Sciences University of Bologna Bologna Italy

**Keywords:** COVID‐19, epidemiology, incidence, Parvovirus B19, pregnancies, seroprevalence

## Abstract

Parvovirus B19 (B19V) is the most relevant human pathogenic virus in the *Parvoviridae* family. In years 2023–2024, a high incidence of B19V infections has been reported from many countries; we reconstructed the circulation of B19V in an Italian metropolitan area during the past 12 years (2012–2024), to elucidate evolving epidemiological trends and the impact of the COVID‐19 pandemic. To this aim, we included a consecutive time‐series analysis of the B19V laboratory investigation carried out in the Microbiology Unit, S.Orsola University Hospital of Bologna, Italy. A total of 29020 serum samples, from July 2012 to June 2024, were tested for the presence of both B19V IgG/IgM antibodies and/or for the presence of B19V DNA. Results were treated in aggregate form, by elaboration of demographic and laboratory data. Data reveal how circulation patterns of B19V have been conditioned by the COVID‐19 pandemic. From 2012 until 2019, alternating phases of lower (years 2012–2014, 2017–2018) or higher (years 2015–2016, 2019) circulation were present, respectively 1.8%–2.6% and 4.7%–4.9% of tested patients. From 2020 to 2023, the lowest incidence of B19V infection was reported, 1.0%–1.3%. An unprecedented increase was observed in the first 6 months of 2024, up to 20.1%, mainly in the 0–10 and 41–50 age groups. In 2024, 53 infections were diagnosed in 115 pregnant women (46.1%). Our data highlight the epidemiological trends in B19V and confirm both the block during the COVID‐19 pandemic and ensuing upsurge in transmission in 2024. The inclusion of B19V in rationally planned screening and diagnostic protocols appears justified in terms of appropriate surveillance and clinical management.

## 1 Introduction

Parvovirus B19 (B19V) is the most relevant human pathogenic virus in the *Parvoviridae* family [1]. Normally transmitted through the respiratory route, B19V has a marked tropism for erythroid progenitor cells in the bone marrow, the only cells capable to support a productive replication and undergoing apoptosis as the consequence. Replication leads to high‐titer viremia and to a systemic phase of infection; hence, other non‐erythroid tissues can be infected, normally in a non‐productive way, and transmission to contacts can occur. The rise of an adaptive immune response contributes to viral clearance, whereas an ineffective immune response may lead to long‐term persistence and chronic infections. B19V maintains a long‐term persistence in disparate tissues, without any sign of an associated productive replication but raising concerns on the possibility of its reactivation [2].

The clinical course of B19V infection is usually biphasic and of variable presentation. In the first phase, manifestations are related to the virus‐induced block in erythropoiesis, resulting in erythroid aplasia and consequent anemia, whose entity and duration depend on the physiological state of the host, the presence of underlying hematological diseases, and the efficacy of the immune response. In the second phase, manifestations are mostly of inflammatory character and manifest in non‐erythroid tissues, including the typical fifth disease in children. Of concern are chronic infections in case of immunodeficient/immunosuppressed patients, and, since the ability of virus to cross the placental barrier, consequences in case of transplacental transmission and fetal infection [3].

Seroprevalence studies indicate a wide circulation, testified by an age‐related increase of prevalence [4]. Perceived as a mild and self‐limited disease, the diagnosis of B19V infection is, in many instances, overlooked or merely based on clinical presentation. This attitude leads to general underreporting of infection rates and circulation of virus in the population, and may be detrimental to prompt diagnosis in case of a more severe impact on the patients. A laboratory diagnosis is thus required for appropriateness, relying on the detection of specific antibodies and viral DNA in peripheral blood or tissues [5].

In the year 2024, a high incidence of B19V infections has been reported from many European countries [6], interpreted as a consequence of the lowest circulation of virus observed during the COVID‐19 pandemic years [7, 8, 9]. Our laboratory, serving an Italian metropolitan area inhabited by more than 900 000 people, has an established experience in the diagnosis of B19V infection. In the present report, we reconstruct local circulation of the virus in the past 12 years (2012–2024), highlighting epidemiological trends and confirming both the block during the COVID‐19 pandemic and the ensuing upsurge in transmission in 2024, and critically evaluating the diagnostic information provided in support of epidemiology surveillance and patient management.

## 2 Materials and Methods

We performed a consecutive time‐series analysis of the Parvovirus B19 laboratory investigation carried out in the Microbiology Unit, IRCSS S.Orsola University Hospital of Bologna, Italy, by using patient‐level electronic health records. The records allowed retrieval of demographic data of patients, date of sampling, material analyzed, and test results. Indications for B19V testing included targeted requests upon clinical suspicion, request in the differential diagnostic work‐up in cases of atypical pathologies, patient follow‐up, screening in the course of pregnancy, or immunosuppressive treatment. All data were available as a result of institutional activity; patients have been treated as anonymous; basic demographic and laboratory data have been treated in aggregate form. Thus, available data could be used under Italian Privacy Law without patient informed consent, however, not allowing access to clinical records. The study was notified and approved by the Local Ethical Committee (661/2023/Oss/AOUBo_VIROSEQ_BO).

We report results obtained from serum samples, tested for the presence of both B19V IgG/IgM antibodies and/or for the presence of B19V DNA, in the period July 2012 to June 2024. Antibodies were detected using chemiluminescence tests (all from DiaSorin, Saluggia, Italy). Assay formats varied: in 2012–2014, Biotrin Parvovirus B19 IgG/IgM EIA, results as index value in the range of 0–12 for IgG and 0–16 for IgM; in 2015–2023, LIAISON Biotrin Parvovirus B19 IgG/IgM, Index Value in the range 0–46 for IgG and 0–48 for IgM; from 2024, LIAISON Biotrin Parvovirus B19 IgG/IgM plus, results as IU/mL in the range 0–150 for IgG and as Index Values in the range 0–48 for IgM. The IgM test claims a diagnostic sensitivity of 99.1% and diagnostic specificity of 99.6%; the IgG test claims a diagnostic sensitivity of 99.8% and diagnostic specificity of 99.5%. For our study, a standard cut‐off set at index value (or IU/mL) 3.0 for both IgG and IgM assured the classification of equivocal results as negative. The genome of B19V (DNA‐B19V) was detected using a quantitative PCR (qPCR) test (B19V ELITe MGB Kit; ELITechGroup, Torino, Italy). The test can detect all three B19V genotypes and claims a detection threshold of 100 copies/mL and a linear range of quantification from 250 to 50 000 000 copies/mL.

Elaboration of numerical data and statistical analysis as appropriate was carried out by GraphPad Prism v.9 (GraphPad Software).

## 3 Results

### 3.1 Samples

From July 2012 to June 2024, a total of 29 020 serum samples have been investigated for the presence of markers of B19V infection, IgG/IgM specific antibodies, and/or DNA. The number of analyzed samples has been constant in the years 2013–2023, with 2351 samples per year on average (range 1992–2640). In 2024, a total of 2200 analyzed samples has been reached within the first 6 months, a likely consequence of an increased awareness of a higher circulation of B19V in the population. Contribution by gender indicates a higher abundance of samples from female, 55.1% versus 44.9 (*p* < 0.0001 by unpaired *t*‐test), constant over years and more significant in 2024, 60.1% versus 39.9% (*p* < 0.0001 by two‐way ANOVA and Bonferroni posttest). Contribution by age indicates a non‐uniform distribution among different groups. A significantly higher abundance of samples was registered in the 0–10 (mean 21.8%, range 12.7%–28.7%), 31–40 (20.3%, 15.5%–26.5%), and > 60 (16.4%, 11.7%–20.9%) age groups, compared to the 11–20 (9.1%, 6.2%–11.2%), 21–30 (9.7%, 7.9%–11.1%), 41–50 (11.3%, 9.6%–13.0%), and 51–60 (11.4%, 9.6%–13.2%) age groups (*p* < 0.0001 by one‐way ANOVA and Dunnett multiple comparison test). Comparing the age group composition among the different years, the only significant shift was the decrease in the 0–10 age group from years 2012–2013 compared to the years 2022–2024 (28.5% vs. 14.5%) (*p* < 0.01 by two‐way ANOVA and Bonferroni multiple comparison test).

**FIGURE 1:**
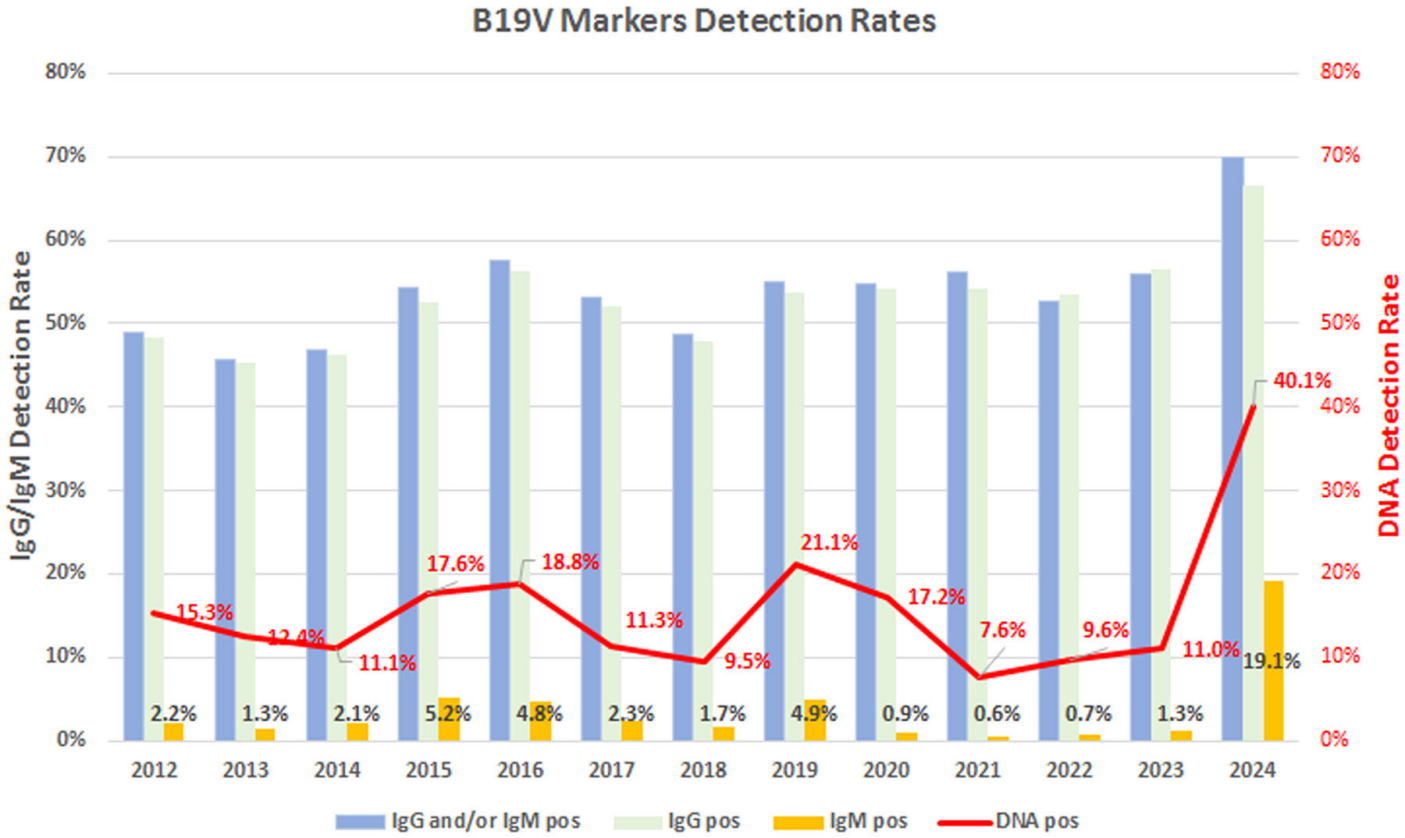
B19V infection markers detection rates. IgG/IgM antibody (left *y*‐axis) and DNA (right *y*‐axis) positivity rates, years 2012–2024.

Of the 29020 samples, 25 981 were analyzed for detection of B19V‐specific IgG/IgM antibodies; 14 016 (53.9%) were positive. IgG was detected in 13 760 (53.0%) samples and IgM in 971 (3.7%) samples. PCR analysis was performed on 4659 samples, and yielded positive results in 768 (16.5%). Antibody detection and PCR positivity through years are reported in Figure 1. Antibody (IgG and/or IgM) positive rates varied between the lower limit of 45.6% recorded in 2013 and the highest limit of 70.0% in the first 6 months of 2024. IgM positivity varied between the lower rate of 0.6% in 2021 to the highest 19.1% in 2024. PCR detection rates varied between a lower limit of 7.6% in 2021 and a higher 40.1% in 2024. All combined, data indicate an evolving pattern of viral circulation. A cyclic pattern of alternating lower (endemic) and higher (epidemic) viral circulation was observed through years 2012–2019, with years 2015–2016 and 2019 showing higher values. In years 2020–2022, coincident with the COVID‐19 pandemic years, the lowest rate of detection was observed. Afterwards, starting in 2023 but strikingly in the first semester of 2024, an unprecedented high rate of B19V infections was observed.

**FIGURE 2:**
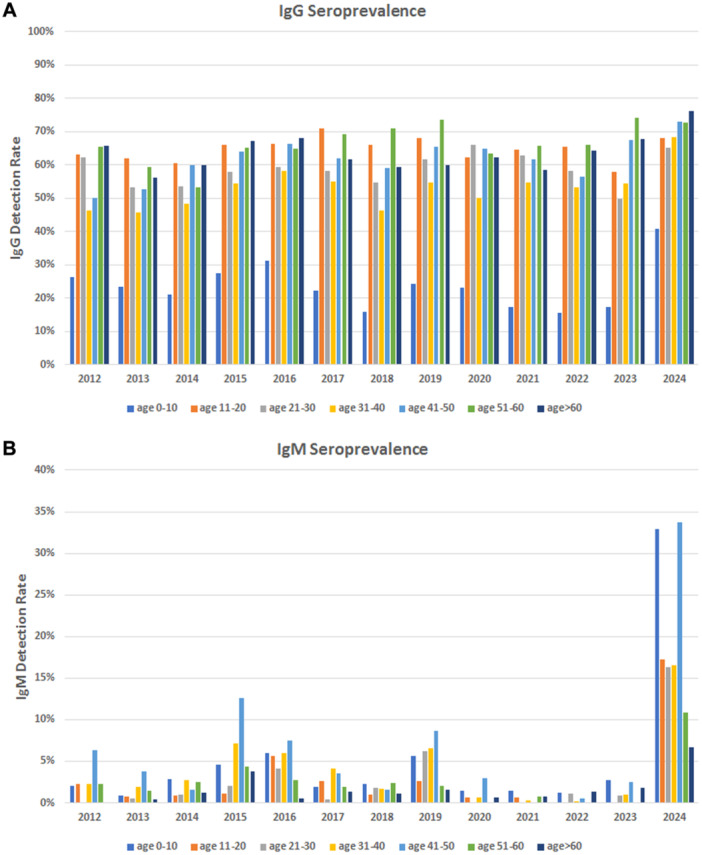
B19V specific IgG and IgM seroprevalence. IgG (A) and IgM (B) antibody detection rates by age groups, years 2012–2024.

IgG positivity rates were comparable during the endemic/epidemic period (years 2012–2019; average 50.3%, range 45.3%–56.4%), the COVID‐19 pandemic years (years 2020–2022; average 54.0%, range 53.6%–54.30%) and the post‐pandemic year 2023 (56.4%) (Figure 1). A significant increase was observed in the first 6 months of 2024 (66.5%). When stratified by age groups (Figure 2A), the 0–10 years group showed a significant lower seroprevalence, 23.5%, compared to the average for all other groups, 60.6% (range 53.0%–66.5%) (*p* < 0.0001 by two‐way ANOVA and Bonferroni posttest). In 2024, an overall increase was observed for all age groups, mainly contributed by the 0–10 age group, 40.8% compared to average 18.3% in 2020–2023 (*p* < 0.01 by two‐way ANOVA and Bonferroni multiple comparison test). High increase, although to a nonsignificant extent, was also observed in the 31–40 age groups, 68.3% compared to average 53.1% in 2020–2023, and by the > 60 age groups, 76.3% compared to average 63.2% in 2020–2023. Considering the nonsignificant variations in the composition of the sample population by age groups in 2024 compared to previous years, the observed variation on IgG seroprevalence testify to the widest circulation of B19V in the first 6 months of 2024, mainly in the younger age group.

IgM positivity rates showed periodicity patterns until 2019, oscillating between high prevalence years (2015, 5.2%; 2016, 4.8%; 2019, 4.9%) and low prevalence years (2012, 2.2%; 2013, 1.3%; 2014, 2.1%; 2017, 2.3%; 2018, 1.7%). Then, a substantial break in the regular circulation of the virus was observed, 0.6%–1.3% in 2020–2023, followed in 2024 by the highest increase ever observed in the analyzed years, 19.1% (Figure 1). A significant increase was observed for all age groups (Figure 2B), highest for the 0–10 (33.0%) and 41–50 (33.7%) groups. Such a high relative rate of IgM detection in the 0–10 group is peculiar of year 2024, compared to previous epidemic years.

**FIGURE 3:**
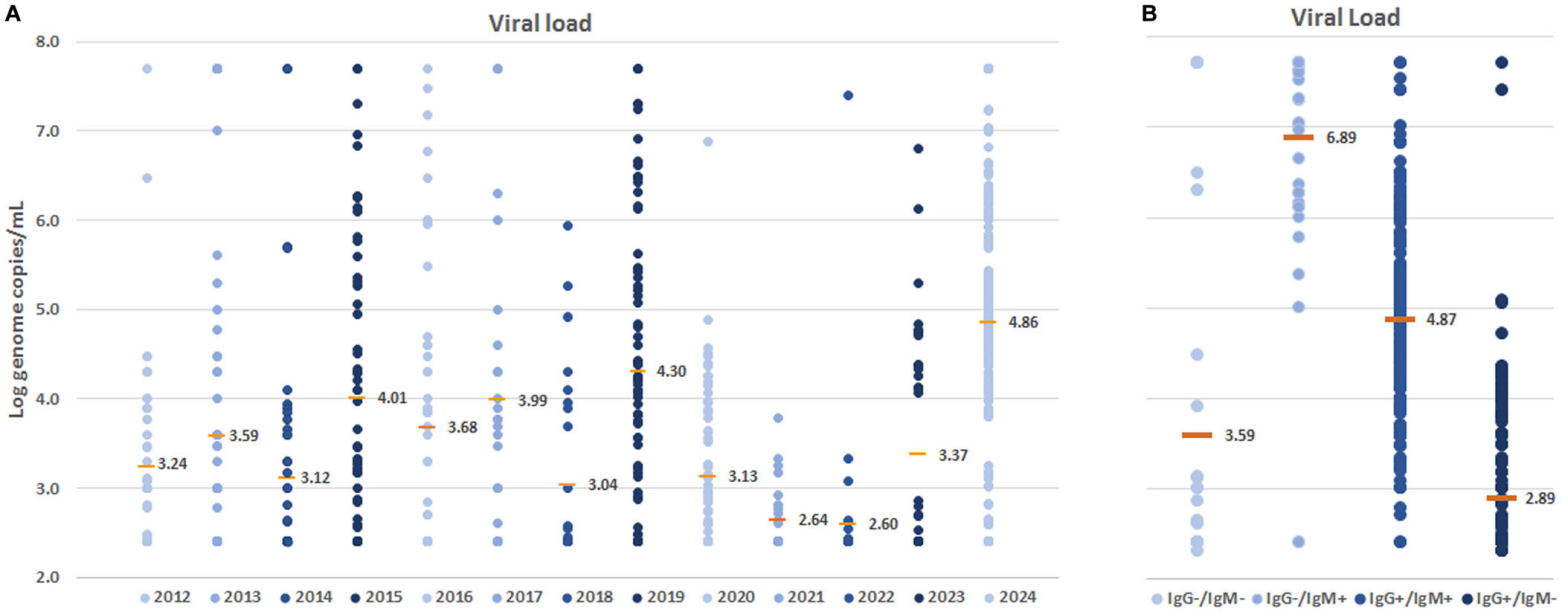
Positive PCR samples viral load, Log genome copies/mL. (A) Individual and mean values, years 2012–2024. (B) Individual and mean values, according to antibody status.

B19V DNA detection rates followed a cyclic pattern of prevalence in the 2012–2019 period (14.6%, range 9.5%–21.1%), showed a marked reduction in the 2020–2023 period (11.3%, range 7.6%–17.2%) and then a substantial increase from January 2024 (40.1%) (Figure 1). Individual viral load values are distributed over the whole dynamic range of molecular test. Average values among the different years conform to a normal distribution (Figure 3A), ranging from a min of 2.60 Logs to a max of 4.86, median 3.36 Logs, interquartile range 3.08–4.00. Higher average values observed in years 2015, 2017, 2019, and 2024 are in correspondence to higher prevalence years, and the lowest values observed in years 2021–2022 correspond to the lowest viral circulation during pandemic. When grouped according to the different combinations of antibody patterns, when known, individual viral loads are widely distributed, but mean viral load values differ significantly: 3.59 Log copies/mL in the IgG−/IgM− (*n* = 35), 6.89 Log copies/mL in the IgG−/IgM+ (*n* = 38), 4.87 Log copies/mL in the IgG+/IgM+ (*n* = 193) and 2.89 in the IgG+/IgM− (*n* = 188) groups (Figure 3B).

**FIGURE 4:**
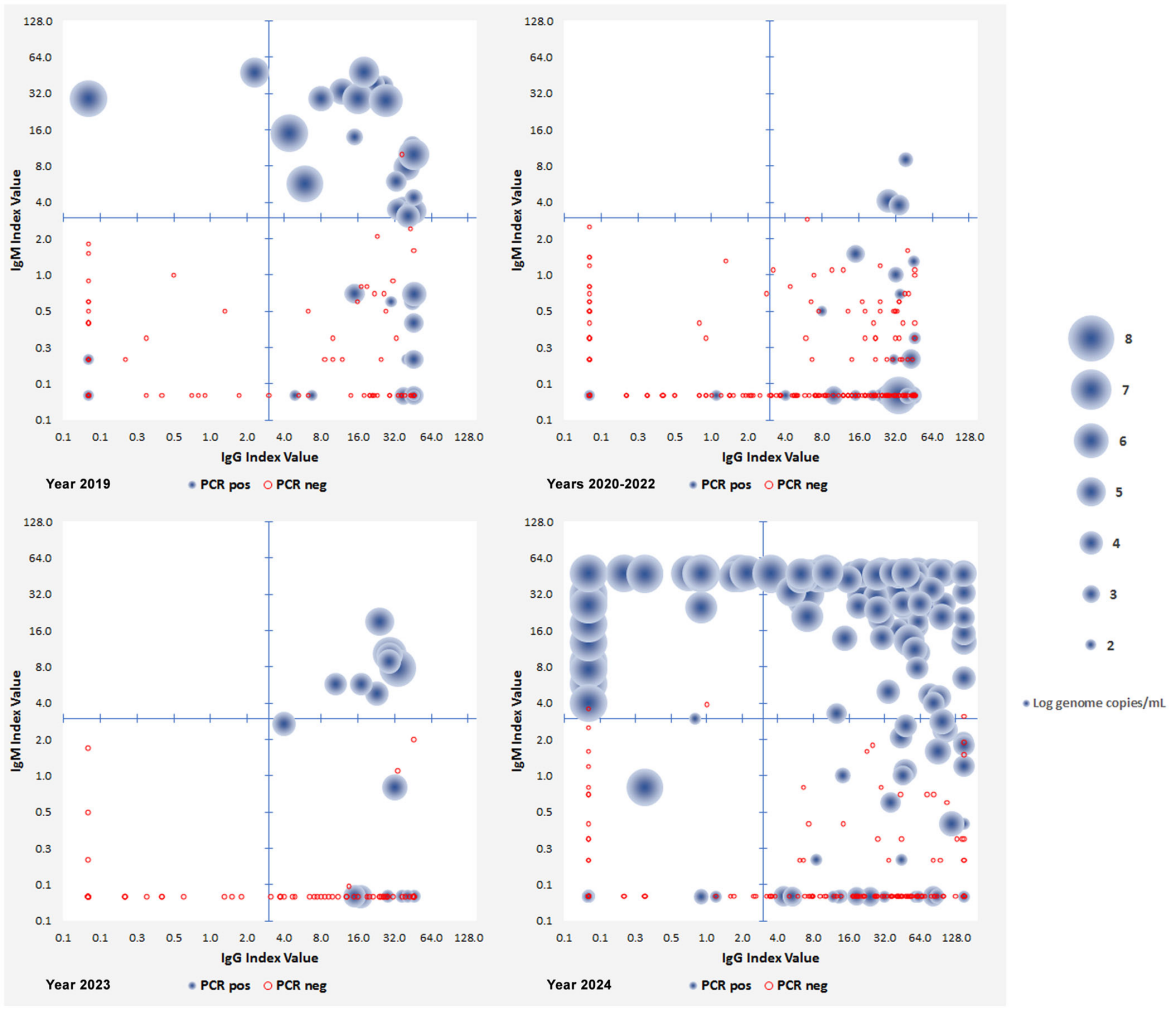
Bubble graph diagrams of B19V infection markers in serum samples, years 2019, 2020–2022, 2023, and 2024. IgG/M titers are reported on the *X*/*Y* axis (Index values, Log_2_ scale), and viral load (Log genome copies/mL) is reported as bubble diameter according to scale.

A comprehensive diagnostic profile, by combined IgG/IgM antibody and PCR analysis, has been obtained on 2117 samples. Of these, 482 samples showed positivity for IgM and/or DNA, 770 were IgG positive only, and 865 showed negative for all markers. PCR positivity provided a unique marker of infection in 38 samples, in 224 it was coupled to IgM presence, while in additional 201 samples it occurred in the presence of IgG only. IgM positivity occurred in 19 samples as the unique marker of infection in the absence of detectable DNA, of whom 12 had IgM only and 7 had both IgM and IgG. Combined results for the years 2019–2024 are reported in Figure 4 as bubble graphs, where each sample is characterized by its position on axis coordinates, related to IgG/IgM antibody titer, and bubble diameter, related to viral load. Samples characterized by high viral load and absence of specific IgM and IgG, a situation observed either in the initial phase of an infection, or in case of profound immunodeficiency/immunosuppression, have been sporadically detected through years. Samples characterized by high viral loads coupled to IgM presence, either with or without IgG, a common finding in case of active/recent infections, have been typically detected in high incidence years (e.g., years 2019, 2023–2024). Samples characterized by lower viral loads, in the presence but sometimes in the absence of IgG, possibly reflecting slowly‐clearing or persistent infections have been observed more frequently in low‐incidence years.

### 3.2 Patients

A 12‐year retrospective analysis of infection incidence per patient was performed; a B19V incidence case was defined by either a positive IgM test or a positive B19V DNA with viral load > 1000 copies/mL. A threshold of 1000 copies/mL has been set to differentiate acute or recent infections, from prolonged detection of viral DNA as a consequence of delayed clearance [10, 11, 12]. From 2012 to 2024, a total of 29 020 samples referred to a set of 25 528 patients. A total of 993 (3.9%) cases of incident B19V infection were identified by the detection of IgM only (647 patients), and/or DNA positivity (346 patients).

**FIGURE 5:**
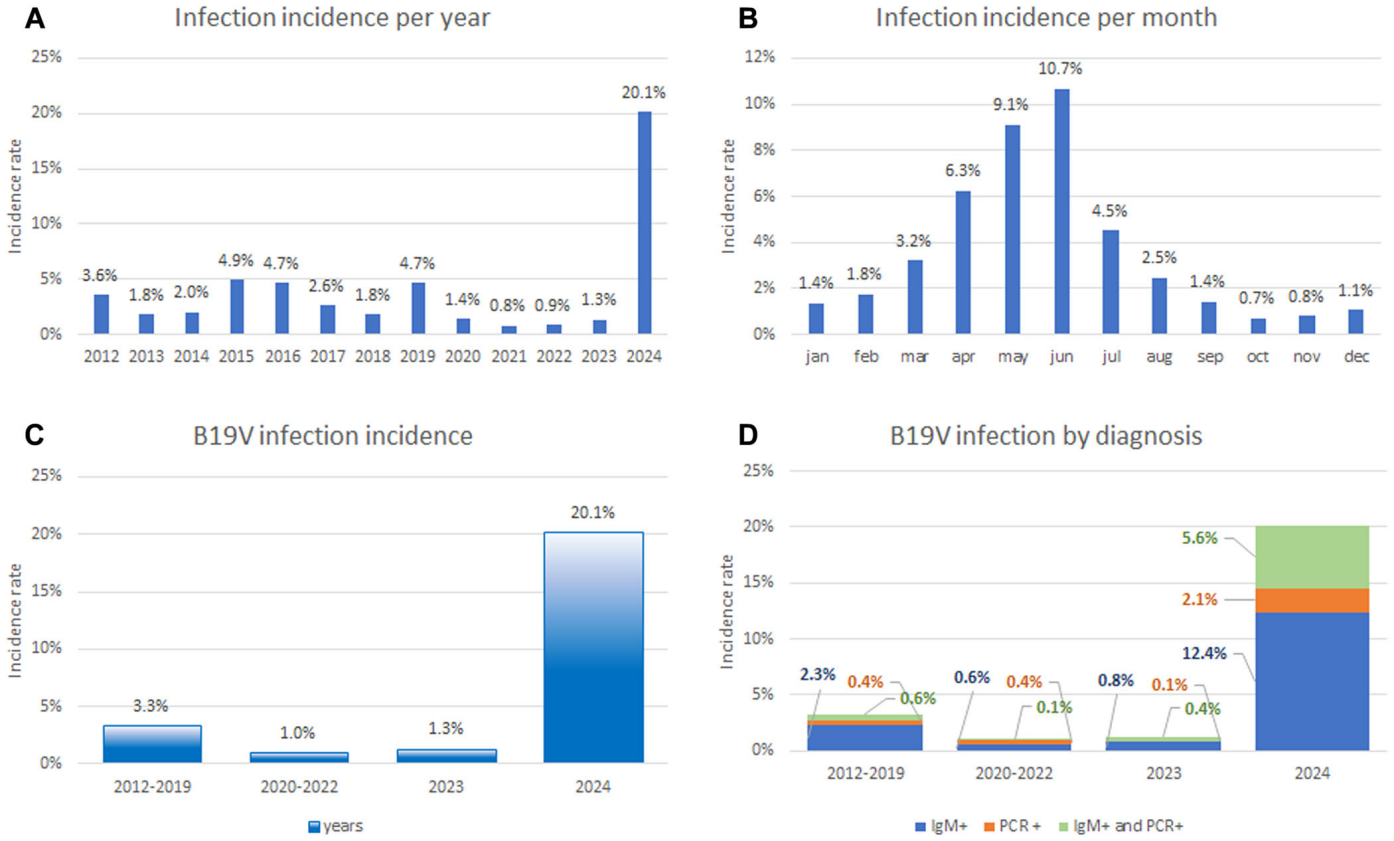
B19V infection incidence per year, 2012–2024 (A), and month (B). B19V infection incidence, total (C) and by diagnosis (D), years 2012–2024.

During the endemic/epidemic years (2012–2019), 535 infected patients were detected out of 16 217 patients screened (3.3%). The highest incidence was confirmed for the years 2015–2016 (4.9%, 4.7%) and 2019 (4.7%). From 2020 to 2022, the lowest incidence of B19V infection was reported: 55 confirmed cases out of 5506 patients tested (1.0%). A moderate increase was observed in 2023, with 25 patients infected with B19V out of 1929 patients tested (1.3%). The highest increase was observed in the first 6 months of 2024 with 378 confirmed B19V infections out of 1876 patients screened (20.1%), statistically significant compared to the pre‐pandemic, pandemic, and 2023 period (*p* < 0.001 by two‐way ANOVA and Bonferroni multiple comparison test) (Figure 5A). Concerning seasonality, no significant variations were found among the different years; infection was mainly diagnosed in late spring—early summer, with 75% of infections occurring from April to July (Figure 5B). In particular, this seasonality pattern presented similar trends in both the pre‐pandemic and post‐pandemic years.

The diagnosis was mainly based on the detection of IgM, 65.2%, with a positivity rate during pre‐pandemic years of 2.3%, interrupted by the COVID‐19 pandemic (0.6% in 2020–2022 and 0.8% in 2023). PCR positivity accounted for 12.8% of diagnosis, and positivity rates were low and stable during pre‐pandemic, pandemic, and in the post‐pandemic 2023 year, respectively, 0.4%, 0.4%, and 0.1%. Combined IgM and PCR detections accounted for 22% of diagnosis; this combination was rare until 2023 (pre‐pandemic, 0.6%; pandemic, 0.1%; post‐pandemic 2023, 0.5%). During the first 6 months of 2024, an unprecedented rebound of viral circulation was documented both with IgM (12.4%) or B19V DNA detection (2.1%), or by the combination of IgM and B19V DNA (5.5%) (Figure 5C,D).

In addition to incidence cases, from 2012 to 2024, 261 patients had a diagnosis of B19V infection based on detection of low‐level DNA (< 1000 copies/mL), without specific IgM, either in the presence or even absence (16.1%) of specific IgG. Thus, low viral loads accounted for 43.0% of PCR diagnosis; this fraction was significantly higher in 2021–2022, reaching 81.4%, and lowest in 2024, at 19.9%. The analytical profile of low‐level DNA, absence of IgM, and variable presence of IgG was mainly interpreted in terms of the initial infection phase with no clinical follow‐up, persistent infections, or mere delayed viral clearance.

### 3.3 In Pregnancy

**FIGURE 6:**
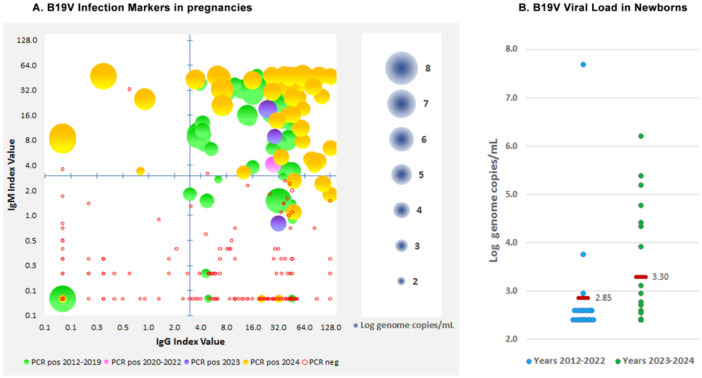
(A) Bubble graph diagrams of B19V infection markers in pregnancies (IgG/M titers on the *X*/*Y* axis, Log genome copies/mL as bubble diameter), in a subset of 313 patients investigated for complete profile, grouped by years. (B) In newborns (< 3 months of age), positive PCR samples viral load, individual and mean values grouped by years.

Data concerning pregnant women afferent to the Maternal‐Fetal Medicine Divisions of hospitals in the metropolitan area were collected. In the whole period, a total of 786 pregnant women were investigated; 114 women resulted infected with B19V. In the pre‐pandemic period 2012–2019, 57 infections were diagnosed in 444 women (12.8%). In 2020–2023, only 4 infections were diagnosed in 227 women (1.8%). In 2024, 53 infections were diagnosed in 115 women (46.1%). Bubble graph distribution of IgG/M and PCR values for a subset of 313 pregnant women for whom a complete analytical profile is available is shown in Figure 6A.

Of 114 B19V‐infected pregnant women, 86 had both DNA (> 1000 copies/mL) and IgM; 15 had DNA without IgM, and 13 had IgM without DNA. Thus, positive IgM or DNA was the unique diagnostic marker in 11.4% and 13.1%, respectively, highlighting the critical need of a combined approach to effective laboratory diagnosis. Out of the 786 pregnancies, further 13 cases (1.7%) were characterized by low‐level viremia (< 1000 copies/mL) in the presence of IgG, possibly referring to low‐level persistence of virus following remote infection or reinfections. A record of newborns aged less than 3 months was also obtained, investigating for the presence of B19V DNA as an indication of congenital infection. Out of 325 newborns, DNA was detected in 25 cases. Viral load distribution is shown in Figure 6B. Of the 25 newborns, 18 showed viral loads < 1000 copies/mL, and only 7 > 1000 copies/mL. Median values differed in years 2023–2024 (*n* = 6), 4.98 Log versus 2.4 Log in years 2012–2022 (*n* = 19), possibly reflecting the higher incidence of virus and increased detection rate.

## 4 Discussion

We present a record of the diagnostic activity related to B19V, carried out in an institutional laboratory serving a whole metropolitan area, Italy, in the period July 2012–June 2024. Indications for B19V testing included targeted requests upon clinical suspicion, request in the differential diagnostic work‐up in cases of atypical pathologies, patient follow‐up, screening in the course of pregnancy, or immunosuppressive treatment. We investigated for the presence of B19V‐specific IgG/M antibodies and/or viral DNA in peripheral blood as markers of infection; the analyzed sample set has not been filtered based on any of further specification. As a limit, our record does not represent a population‐based survey of circulation of virus in the area. On the other hand, our data provide a full record of consecutive laboratory diagnostic activity, allowing informed inference on the circulation of virus in the area and on its clinical impact.

The analytical techniques have been consistent throughout years, except for limited technical upgrades. Diagnosis, fully integrated into automated laboratory workflow, relied on the coupled detection of specific anti‐B19V antibodies of both IgG and IgM classes, and/or on the quantitative detection of viral DNA in peripheral blood, using serum as the standard material. In both cases, results are quantitative; for antibodies, reactivities are expressed in terms of index values, while for B19V DNA quantitative results are expressed as copies/mL. In addition to positivity rates, quantitative evaluation of antibody titer and viral load better contribute to the definition of infection status, relating viremia and immune system response. Overall, 83.9% of samples have been analyzed for the presence of specific antibodies only, 10.5% have been analyzed for the detection of viral DNA only, and a mere 5.6% was investigated for a combined profile, including specific antibodies and viral DNA. Hence, information from this subset clearly shows how a correct diagnosis is best obtained when antibodies and quantitative DNA detection are coupled. Antibody detection remains a mainstay for investigation in a large number of samples, but as the only diagnostic tool, it may lead to underestimation of active infections, not only for acute phase infections in the seroconversion phase, but chiefly in cases of chronic infections. Direct information on the presence of viral DNA as indicative of active infections can be provided by quantitative PCR; however, since individual viral levels do not directly correlate with the patient serostatus, information on the infection course can be incomplete. Therefore, it is the combined detection and quantitative evaluation of antibodies and viral DNA that enables the definition of a complete analytical profile, more appropriate to characterize the dynamics of the infectious process and host response. Bubble graphs are an effective means of conveying this combined information.

On a population level, both per‐sample and per‐patient records clearly indicate circulation of B19V in the area, with different evolving patterns. From 2012 until 2019, circulation conformed to the typical endemic/epidemic cycles, alternating phases of lower (years 2012–2014, 2017–2018) or higher (years 2015–2016, 2019) circulation. The SARS‐CoV2 pandemic imposed dramatic consequences on B19V circulation, considering the very stringent lockdown enforcement in our metropolitan area and the diffuse use of personal protection devices lasting until 2022. As a result, in face of a mere 25% decrease in the number of analyzed samples at the height of pandemic, B19V disappeared from records in a sudden and almost total degree, the few positive samples being likely attributed to persistent infections. This situation held until early 2023, heralding the sharpest increase observed in 2024, unprecedented in our metropolitan area. Indirectly, this higher circulation is confirmed by the rate of seropositive samples, rising from 56% to 70%, unrelated to any significant change in demography and sample population compositions compared to the previous years. Such general increase was contributed from all age groups, and in particular, from 0 to 10 and 41 to 50 groups, suggesting intense transmission within school and household environments.

Our data align with already available data. In the period 2020–2024, analysis of viremic blood donations first anticipated the block in circulation [13], then confirmed the rebound in 2023–2024 [14, 15, 16, 17], including Italy [18]. An increased circulation of B19V was reported in 2023 in France [19], Netherlands [20], and Israel [21, 22], followed in 2024 by additional national surveillance reports, leading to an ECDC brief on B19V issued in June 2024 [6] and a CDC alert in August 2024 (CDC Health Alert network CDCHAN‐00514). Our experience, by expanding comparison in a 12‐year period, highlights the different trends in epidemiological patterns of B19V through years and confirm the impact of the COVID‐19 pandemic on its circulation. Similar to what has been observed for other respiratory viruses, the block coincident with the onset of the COVID‐19 pandemic can be ascribed to the general nonmedical countermeasures adopted. Peculiar to B19V has been the long time to reintroduction in the community, from 2020 lagging until 2024, and conversely the intense ensuing epidemic, locally resulting in a total 14% increase of seroprevalence in the sampled population, and a 30‐fold increase in the number of diagnosis of acute infections. As a term of comparison, the fraction B19V‐positive blood units collected for manufacturing in Italy (all regions) in the first 6 months of 2024 was 36.7 per 100 000, peaking at 59.4 in March and April, and declining afterwards [18].

Overall, B19V infection remains overlooked and underdiagnosed in the population. Even during the 2024 epidemic, a laboratory diagnosis of incident infection was obtained in 378 cases, 20.1% of 1876 investigated patients, themselves a mere 0.2% sampling on the population in the metropolitan area, in front of a 14% increase in the seroprevalence from 2023 to 2024. Such underrepresentation of B19V in the medical record conforms to a consolidated general attitude toward a virus that is considered of little clinical concern. Actually, severe or atypical presentations are prone to be missed when B19V is not included in standardized diagnostic protocols, as in myocarditis [23]. Increased circulation of the virus increases the frequency of atypical clinical manifestations, the risk for immunocompromised subjects, including transplanted patients, and the risks related to maternal‐fetal transmission, also reported at increased frequency in 2024 [24, 25, 26, 27]. The inclusion of B19V in rationally planned screening and diagnostic protocols appears justified in terms of appropriate surveillance and clinical management. A special focus of concern should be maintained for the risks related to maternal‐fetal transmission. Epidemiological models referred to European context predict that about 1% of pregnant women are at risk of acquiring primary B19V infection [4], with an estimated rate of transplacental transmission of about 33%, and of fetal demise up to 10% [3, 28]. In this subset of patients, the number of analyzed samples and, hence, the incidence of detected B19V infections can vary greatly in the years, mostly according to the perception of risk. From our record, it emerges that the amount of B19V diagnostic activity related to pregnancies is, however, insufficient to correctly monitor the incidence of infections and possible consequences on the fetuses and newborns. Concerning the outbreak in 2024, the high number of infections in pregnancies, and the high number of congenital infections characterized by a relatively high viral load at birth, will offer a valuable opportunity of extended investigation, to reassess the impact of B19V in pregnancies, and the consequences of infection in the newborns.

The high circulation of B19V observed in 2024 as a consequence of the break during the SARS‐CoV2 pandemic was a predictable event, providing the opportunity for interesting comparison among different epidemiological trends, and prompting for increased clinical awareness and diagnostic preparedness. While B19V is not considered a potential pandemic threat [29], it is a widely circulating virus capable of sustained epidemics, with an ample and diverse pathogenic potential. In the near future, given the high incidence of the virus, and the still relatively high fraction of the immunological naïve population, it cannot be excluded a sustained viral circulation before returning to the more contained fluctuations typically observed until 2019. Ongoing research to understand B19V biological properties, its pathogenic potential, and the development of effective antiviral strategies [30], coupled to a proactive diagnostic and therapeutic approach, is warranted.

## Author Contributions

Data collection and elaboration were carried out by Simona Venturoli, Alessia Bertoldi, and Elisabetta Manaresi. Data analysis was conducted by Simona Venturoli, Tiziana Lazzarotto, and Giorgio Gallinella. Manuscript preparation was handled by Simona Venturoli, Elisabetta Manaresi, and Giorgio Gallinella.

## Conflicts of Interest

The authors declare no conflicts of interest.

## Supporting information

Supporting information.

## Data Availability

The data that support the findings of this study are available on request from the corresponding author. The authors have nothing to report.
