# Supplementary material for: Epidemiology of Parvovirus B19 Infection In an Italian Metropolitan Area, 2012–2024: COVID‐19 Pre‐Pandemic, Pandemic and Post‐Pandemic Trends
Source: J Med Virol. 2025 Mar 17;97(3):e70296. doi: 10.1002/jmv.70296 (PMC11911903; doi:10.1002/jmv.70296)
Supplement: Supplementary file 1 — Supporting information. [file JMV-97-e70296-s001.docx]

**Table 1**. Serum samples collected between 07/2012 and 06/2024 and included for analysis in the present study.

| **Year** | **total** | **gender** | | **age 0-10** | | **age 11-20** | | **age 21-30** | | **age 31-40** | | **age 41-50** | | **age 51-60** | | **age > 60** | | **total** |
| --- | --- | --- | --- | --- | --- | --- | --- | --- | --- | --- | --- | --- | --- | --- | --- | --- | --- | --- |
|  | **n** | **male %** | **female %** | **n** | **%** | **n** | **%** | **n** | **%** | **n** | **%** | **n** | **%** | **n** | **%** | **n** | **%** | **n** |
| **2012** | 956 | 48.1% | 51.9% | 274 | 28.7% | 96 | 10.0% | 76 | 7.9% | 148 | 15.5% | 123 | 12.9% | 114 | 11.9% | 125 | 13.1% | 956 |
| **2013** | 2368 | 46.8% | 53.2% | 673 | 28.4% | 266 | 11.2% | 204 | 8.6% | 442 | 18.7% | 261 | 11.0% | 245 | 10.3% | 277 | 11.7% | 2368 |
| **2014** | 2572 | 45.6% | 54.4% | 671 | 26.1% | 246 | 9.6% | 226 | 8.8% | 504 | 19.6% | 297 | 11.5% | 270 | 10.5% | 358 | 13.9% | 2572 |
| **2015** | 2633 | 46.8% | 53.2% | 688 | 26.1% | 279 | 10.6% | 228 | 8.7% | 483 | 18.3% | 297 | 11.3% | 294 | 11.2% | 364 | 13.8% | 2633 |
| **2016** | 2640 | 43.5% | 56.5% | 609 | 23.1% | 276 | 10.5% | 263 | 10.0% | 478 | 18.1% | 338 | 12.8% | 269 | 10.2% | 407 | 15.4% | 2640 |
| **2017** | 2275 | 46.5% | 53.5% | 557 | 24.5% | 158 | 6.9% | 227 | 10.0% | 409 | 18.0% | 245 | 10.8% | 281 | 12.4% | 398 | 17.5% | 2275 |
| **2018** | 2337 | 43.1% | 56.9% | 538 | 23.0% | 217 | 9.3% | 251 | 10.7% | 512 | 21.9% | 224 | 9.6% | 225 | 9.6% | 370 | 15.8% | 2337 |
| **2019** | 2617 | 43.0% | 57.0% | 612 | 23.4% | 207 | 7.9% | 273 | 10.4% | 492 | 18.8% | 298 | 11.4% | 292 | 11.2% | 443 | 16.9% | 2617 |
| **2020** | 1992 | 45.1% | 54.9% | 386 | 19.4% | 210 | 10.5% | 183 | 9.2% | 350 | 17.6% | 226 | 11.3% | 263 | 13.2% | 374 | 18.8% | 1992 |
| **2021** | 2093 | 45.3% | 54.7% | 362 | 17.3% | 189 | 9.0% | 211 | 10.1% | 417 | 19.9% | 219 | 10.5% | 271 | 12.9% | 424 | 20.3% | 2093 |
| **2022** | 2142 | 44.7% | 55.3% | 340 | 15.9% | 182 | 8.5% | 212 | 9.9% | 520 | 24.3% | 215 | 10.0% | 250 | 11.7% | 423 | 19.7% | 2142 |
| **2023** | 2195 | 44.9% | 55.1% | 280 | 12.8% | 137 | 6.2% | 245 | 11.2% | 572 | 26.1% | 224 | 10.2% | 278 | 12.7% | 459 | 20.9% | 2195 |
| **2024** | 2200 | 39.9% | 60.1% | 324 | 14.7% | 186 | 8.5% | 228 | 10.4% | 584 | 26.5% | 287 | 13.0% | 241 | 11.0% | 350 | 15.9% | 2200 |
| **total** | **29020** |  |  | **6314** |  | **2649** |  | **2827** |  | **5911** |  | **3254** |  | **3293** |  | **4772** |  | **29020** |

**Table 2**. Distribution of B19V infection markers in the sample population, years 2012-2024.

| **Year** | **total Ig** | **IgG and IgM neg** | | **IgG and/or IgM pos** | | **IgG pos** | | **IgM pos** | | **total PCR** | **DNA pos** | |
| --- | --- | --- | --- | --- | --- | --- | --- | --- | --- | --- | --- | --- |
|  | **n** | **n** | **%** | **n** | **%** | **n** | **%** | **n** | **%** | **n** | **n** | **%** |
| 2012 | 812 | 415 | 51.1% | 397 | 48.9% | 393 | 48.4% | 18 | 2.2% | 281 | 43 | 15.3% |
| 2013 | 2104 | 1144 | 54.4% | 960 | 45.6% | 954 | 45.3% | 28 | 1.3% | 539 | 67 | 12.4% |
| 2014 | 2317 | 1233 | 53.2% | 1084 | 46.8% | 1070 | 46.2% | 48 | 2.1% | 414 | 46 | 11.1% |
| 2015 | 2362 | 1080 | 45.7% | 1282 | 54.3% | 1243 | 52.6% | 122 | 5.2% | 340 | 60 | 17.6% |
| 2016 | 2502 | 1060 | 42.4% | 1442 | 57.6% | 1410 | 56.4% | 119 | 4.8% | 261 | 49 | 18.8% |
| 2017 | 2144 | 1003 | 46.8% | 1141 | 53.2% | 1117 | 52.1% | 49 | 2.3% | 240 | 27 | 11.3% |
| 2018 | 2123 | 1091 | 51.4% | 1032 | 48.6% | 1015 | 47.8% | 37 | 1.7% | 294 | 28 | 9.5% |
| 2019 | 2355 | 1057 | 44.9% | 1298 | 55.1% | 1266 | 53.8% | 116 | 4.9% | 355 | 75 | 21.1% |
| 2020 | 1701 | 769 | 45.2% | 932 | 54.8% | 923 | 54.3% | 15 | 0.9% | 379 | 65 | 17.2% |
| 2021 | 1860 | 814 | 43.8% | 1046 | 56.2% | 1007 | 54.1% | 11 | 0.6% | 329 | 25 | 7.6% |
| 2022 | 1832 | 866 | 47.3% | 966 | 52.7% | 982 | 53.6% | 12 | 0.7% | 375 | 36 | 9.6% |
| 2023 | 1923 | 849 | 44.1% | 1074 | 55.9% | 1085 | 56.4% | 25 | 1.3% | 326 | 36 | 11.0% |
| 2024 | 1946 | 584 | 30.0% | 1362 | 70.0% | 1295 | 66.5% | 371 | 19.1% | 526 | 211 | 40.1% |
| **Total** | **25981** | **11965** |  | **14016** |  | **13760** |  | **971** |  | **4659** | **768** |  |

**Table 3**. Distribution of B19V infection markers per patient, years 2012-2024. Total incident infections are given by the sum of patients with PCR >1000 copies/mL, and IgM positivity in the absence of PCR data.

| **year** | **Tot PCR pos** | **>1000 UI/mL** | **<1000 UI/mL** | **IgM+ PCR+** | **IgM+** | **Tot Infections** | **Tot patients** | **Incidence** |
| --- | --- | --- | --- | --- | --- | --- | --- | --- |
| **2012** | 34 | 19 | 15 | 4 | 12 | 31 | 862 | 3.6% |
| **2013** | 49 | 23 | 26 | 11 | 15 | 38 | 2105 | 1.8% |
| **2014** | 36 | 15 | 21 | 10 | 30 | 45 | 2244 | 2.0% |
| **2015** | 50 | 32 | 18 | 23 | 78 | 110 | 2224 | 4.9% |
| **2016** | 40 | 23 | 17 | 19 | 90 | 113 | 2415 | 4.7% |
| **2017** | 21 | 13 | 8 | 7 | 40 | 53 | 2022 | 2.6% |
| **2018** | 22 | 7 | 15 | 3 | 30 | 37 | 2025 | 1.8% |
| **2019** | 54 | 37 | 17 | 25 | 71 | 108 | 2320 | 4.7% |
| **2020** | 38 | 13 | 25 | 2 | 12 | 25 | 1769 | 1.4% |
| **2021** | 21 | 4 | 17 | 0 | 10 | 14 | 1860 | 0.8% |
| **2022** | 33 | 6 | 27 | 1 | 10 | 16 | 1877 | 0.9% |
| **2023** | 28 | 9 | 19 | 8 | 16 | 25 | 1929 | 1.3% |
| **2024** | 181 | 145 | 36 | 105 | 233 | 378 | 1876 | 20.1% |
| **total** | **607** | **346** | **261** | **218** | **647** | **993** | **25528** | **3.9%** |

**Table 4**. B19V infection in pregnancy and newborns.

| **year** | **pregnant** | **Infections** | **PCR+** | **IgM+ PCR+** | **IgM+** | **Incidence** | **newborns** | **PCR+** | **% PCR pos** |
| --- | --- | --- | --- | --- | --- | --- | --- | --- | --- |
| **2012** | 42 | 5 | 2 | 2 | 1 | 11.9% | 22 | 2 | 9.1% |
| **2013** | 33 | 5 | 0 | 5 | 0 | 15.2% | 46 | 2 | 4.3% |
| **2014** | 51 | 7 | 1 | 5 | 1 | 13.7% | 20 | 1 | 5.0% |
| **2015** | 35 | 1 | 0 | 0 | 1 | 2.9% | 24 | 3 | 12.5% |
| **2016** | 86 | 16 | 2 | 11 | 3 | 18.6% | 26 | 3 | 11.5% |
| **2017** | 62 | 9 | 1 | 6 | 2 | 14.5% | 17 | 1 | 5.9% |
| **2018** | 53 | 3 | 1 | 1 | 1 | 5.7% | 33 | 2 | 6.1% |
| **2019** | 82 | 11 | 0 | 11 | 0 | 13.4% | 37 | 4 | 10.8% |
| **2020** | 61 | 1 | 1 | 0 | 0 | 1.6% | 24 | 0 | 0.0% |
| **2021** | 55 | 0 | 0 | 0 | 0 | 0.0% | 25 | 0 | 0.0% |
| **2022** | 42 | 0 | 0 | 0 | 0 | 0.0% | 24 | 1 | 4.2% |
| **2023** | 69 | 3 | 1 | 2 | 0 | 4.3% | 18 | 1 | 5.6% |
| **2024** | 115 | 53 | 6 | 43 | 4 | 46.1% | 11 | 5 | 45.5% |
| **total** | **786** | **114** | **15** | **86** | **13** | **14.5%** | **327** | **25** | **7.6%** |
